# Perceived vulnerability to disease, knowledge about COVID-19, and changes in preventive behavior during lockdown in a German convenience sample

**DOI:** 10.1007/s12144-021-01456-6

**Published:** 2021-02-26

**Authors:** Ulrich Stangier, Schahryar Kananian, Johanna Schüller

**Affiliations:** 1 Goethe-University Frankfurt, Frankfurt, Germany; 2 Clinical Psychology and Psychotherapy Department of Psychology, Goethe University, Varrentrappstr. 40-42, 60486 Frankfurt am Main, Germany

**Keywords:** COVID-19 pandemic, Fear of infection, Germ aversion, Preventive behavior, Risk behavior

## Abstract

The COVID-19 pandemic has called worldwide for strong governmental measures to contain its spread, associated with considerable psychological distress. This study aimed at screening a convenience sample in Germany during lockdown for perceived vulnerability to disease, knowledge about COVID-19, symptoms of depression and anxiety, and behavioral responses. In an online survey, 1358 participants completed the perceived vulnerability to disease scale (PVD), the Patient Health Questionnaire (PHQ-4), and questionnaires on knowledge about COVID-19 and self-perceived change in behaviors in response to COVID-19. Lower and upper quartiles of the PVD were used to classify individuals into low and high PVD. A confirmatory factor analysis supported three factors representing risk, preventive and adaptive behavior as behavioral responses to COVID-19 lockdown. A structural equation model showed that the score of the knowledge scale significantly predicted the self-reported increase in adaptive and preventive behavior. The score in the PVD-subscale Perceived Infectability predicted a self-reported increase in preventive behavior, whereas the Germ Aversion score predicted a self-reported increase in preventive and a decrease in risk behavior. The score in PHQ-4 predicted a higher score in the perceived infectability and germ aversion subscales, and a self-reported decrease in adaptive behavior. Low-, medium- and high-PVD groups reported distinct patterns of behavior, knowledge, and mental health symptoms. This study shows that perceived vulnerability to disease is closely linked to preventive behaviors and may enhance adaptation to COVID-19 pandemic.

## Introduction

The worldwide spread of the COVID-19 and the subsequent lockdown response in many countries has caused significant emotional distress. There is an increasing number of studies showing that a large proportion of the general population experience considerable symptoms of anxiety, depression, and posttraumatic stress symptoms in China (Qiu et al., 2020), Italy (Mazza et al., 2020), Spain (Gómez-Salgado, Andrés-Villas, Domínguez-Salas, Díaz-Milanés, & Ruiz-Frutos, 2020; Odriozola-González, Planchuelo-Gómez, Irurtia, & Luis-García, 2020), India (Varshney, Parel, Raizada, & Sarin, 2020), Germany (Petzold et al., 2020) and United States of America and Canada (Taylor et al., 2020a, b).

Besides the economic threats, a large part of emotional distress can be explained by fear of infection. The lack of immunity and vaccines against the virus, its exponential spread and high mortality, and the uncertainties concerning etiology and course, contribute to the perception of a largely uncontrollable and unpredictable threat (Asmundson & Taylor, 2020; Taylor et al., 2020a). Furthermore, although compliance with social distancing contributes to the control of the transmission and reduces fear of infection (Milman, Lee, & Neimeyer, 2020), imposed social isolation causes considerable psychological strain and triggers a variety of psychological problems, including depression, loneliness and anger (Brooks, Webster, Smith, et al., 2020; Smith et al., 2020).

In the absence of effective medical interventions and lack of protective immunity, individual’s hygiene behavior including washing hands, wearing masks and avoiding contact, has become a major target of preventive measures. Based on scientific data, recommendations of national authorities for health prevention and disease control aimed at increasing the knowledge of the general population to support preventive behavior. There is much evidence that knowledge about COVID-19 is associated with behavioral changes (Clements, 2020; Wang et al., 2020). In addition, emotional factors such as fear of infection may also influence preventive behavior. However, the data from recent studies are inconsistent, with some studies finding a positive relationship between anxiety, knowledge and preventive behavior (Liu, Luo, Li, et al., 2020; Lei et al., 2020), whereas other studies failed to find any relationship (Wang et al., 2020) or found a negative relationship (Jungmann & Witthöft, 2020).

A general concept regarding health behavior and behavioral change is the Health Belief Model (Rosenstock, Strecher, & Becker, 1988; Jones et al., 2014), which postulates behavioral change as a complex process that involves multiple parameters, such as perceived susceptibility, perceived severity, health motivation, perceived benefits, and perceived barriers. With respect to the COVD-19 pandemic, the behavioral response to the specific threat may also be influenced by the perception of susceptibility to infection. The more specific concept of the behavioral immune system (Schaller & Park, 2011; Taylor, 2019) explains fear of infection and avoidance of related sources as an evolutionary rooted motivation which increases the probability of survival. Derived from that model is the perceived vulnerability to disease (Asmundson & Taylor, 2020; Taylor 2020a), a dimensional measure that may explain individual differences in the reaction to COVID-19 pandemic. Emotional and behavioral reactions in response to the threat of pandemic infections may be associated to the perception of personal risk to be infected, as well as aversion and discomfort in situations associated with increased risk of infection. Using the perceived vulnerability to disease scale (PVDS; Duncan, Schaller, & Park, 2009), significant correlations were found of the total score as well as both subscales, Perceived Infectability and Germ Aversion, with measures of health anxiety and hypochondriasis (Duncan et al., 2009; Díaz, Soriano, & Beleña, 2016), neuroticism (Duncan et al., 2009), negative attitudes towards people with HIV infection (Magallares, Fuster-Ruiz De Apodaca, & Morales, 2017) and towards people from East Asia during the COVID-19 pandemic (Goh, 2020).

The primary aim of our study was to investigate the impact of Perceived Infectability, Germ Aversion and knowledge about COVID-19 on self-reported changes in preventive, adaptive and risk behaviors during lockdown in Germany. We expected that these factors are associated with increased preventive and decreased risk behavior. Furthermore, we were interested whether Perceived Infectability and Germ Aversion mediate the relation of general distress with behavioral responses to the COVID-19 pandemic. Finally, we examined whether subgroups of individuals with different levels of PVD differed regarding behavioral changes, knowledge and emotional distress.

## Method

### Participants and Data Collection Procedures

Data were collected between March 24 and April 28, 2020. The study was approved by the research ethics board of the Department of Psychology, Goethe University Frankfurt. Participants were recruited via the internet, local newspapers, TV and radio appearances. No payment was given for participation. Prior to participation, informed consent was provided. Individuals who tested positive for COVID-19 were not included in the study. The sample comprised 1358 individuals aged 17–78 years (*M* = 41.5 years, *SD* = 15.0). 1040 were female, 309 male, and 9 “other” gender. 15.9% had a high school diploma, 25.5% a Bachelor’s degree, and 57.3% a Master’s degree. 395 participants lived alone, 442 with their partner, 434 with their family, and 87 in shared apartments. 132 respondents (9.7%) reported a current mental health diagnosis, most often depression (5.5%) and posttraumatic stress disorder (1.6%). On the basis of the cut off values for the Patient Health Questionnaire-4 (Löwe et al., 2010), 57% of the sample had elevated levels of depression and anxiety.

### Measures

#### Perceived Vulnerability to Disease (PVD; Duncan et al., 2009)

The PVD is a 15-item self-report questionnaire which measures worries about contagious diseases. Factor analyses suggested two dimensions (Díaz et al., 2016): perceived infectability and germ aversion. Items are scored on a 7-point Likert scale, with endpoints labelled as “strongly disagree” and “strongly agree”. The PVD has performed well on tests of reliability and validity (Duncan et al., 2009; Díaz et al., 2016; Magallares et al., 2017; Diaz, Beleña, & Zueco, 2020). In the present study, two items were excluded which appeared to be no longer appropriate to contemporary life conditions (Item 4: write with a pencil someone else has obviously chewed on; Item 15: avoid using public telephones because of the risk that I may catch something from the previous use). In line with previous studies (Díaz et al., 2016), in this study the internal consistency of the subscale perceived infectability was excellent (*ω* = .91 [95% CI: .90; .92]), but not adequate for the germ aversion subscale (*ω* = .50 [95% CI: .45; .55]). For the analyses, we included the PVD subscales as latent factors in a structural equation model, to take low reliability in the measure into account and adequately correct for it (Hoyle & Smith, 1994). Through latent modelling of the construct, only the reliable portion of eachs item’s variance is taken into account for the formation of the construct and the subsequent regressions, thus increasing the reliability of the regression.

#### Patient Health Questionnaire-4 (PHQ-4; Kroenke, Spitzer, Williams, & Löwe, 2009)

The PHQ-4 is a brief screening scale for anxiety and depression. It comprises four items which refer to common symptoms of anxiety and depression (two items each). The items are scored on a 4-point Likert scale, ranging from 0 (not at all) to 3 (nearly every day). The PHQ-4 has been validated in clinical samples (Kroenke et al., 2009) as well as in a large general population sample (Löwe et al., 2010). In the present study, Cronbach’s alpha for the PHQ-4 was sufficient (α = .87).

#### COVID-19 Knowledge Questionnaire (CKQ)

A questionnaire was developed using the frequently asked questions posted on the websites of the World Health Organization (WHO, 2020) and the German Federal Ministry of Health (Robert-Koch-Institut, 2020). A multiple-choice test was constructed where the respondents were asked to choose a limited set of answers from a list of choices. Distractors were derived from common misinformation on COVID-19 addressed on the websites of the WHO and German health authorities. The term COVID-19 was replaced by “corona infection” as this is the term commonly used to refer to the syndrome in the German population. The questionnaire contained seven questions, one question each on disease epidemiology (3 correct answers out of 6), symptoms (5/10), incubation period (1/3), mode of transmission (5/8), fatality rate (1/4), risk factors (4/6) and preventive strategies (4/7). The final questionnaire was reviewed by three medical doctors for face validity. Knowledge about COVID-19 was quantified as percentage of correct answers, averaged over all seven questions.

#### COVID-19 Behavior Checklist (CBC)

To assess behavioral reactions to COVID-19 lockdown, a checklist of behaviors was composed comprising commonly discussed practices of hygiene (3 items), social activities associated with physical contact (3 items) or social distance (1 item), and health-related activities (4 items). The respondents were asked how the frequency of showing these behaviors changed since the beginning of the pandemic on a 7-point Likert scale, ranging from −3 (“much less”) to +3 (“much more frequent”), with 0 representing “unchanged”.

Based on theoretical considerations, the behavioral items were assigned to the three subscales risk behavior, preventive behavior and adaptive behavior, and this structure was tested with confirmatory factor analysis. In large samples, the *χ*^2^-value is often bloated; therefore the model fit was assessed using only the descriptive fit indices. The resulting model showed a subpar fit (*RMSEA* = 0.065, *SRMR* = 0.060, CFI = 0.940). Based on modification indices for this model, one item (“gathering information from the internet”) was reassigned from the subscale *Adaptive Behavior*, which represents coping with the social restrictions, to *Preventive* Behavior which refers to the hygiene measures. The resulting model showed an acceptable fit (*RMSEA* = 0.056, *SRMR* = 0.052, CFI = 0.955). Resulting factor loadings are given in the Supplementary Material. McDonald Omega for the three subscales ranged from very good (Risk Behavior: *ω* = .88 [95% CI: .87; .89]) to inadequate (Preventive Behavior: *ω* = .45 [95% CI: .40; .50], Adaptive Behavior: *ω* = .56 [95% CI: .51; .61]), indicating low item-intercorrelations. The low internal consistencies of two subscales result from the use of a small number of nonredundant indicators covering a broad range of content (Stanley & Edwards, 2016). However, since the CFA indicated a good model fit and lack of reliability is compensated by using latent factors in a structural equation model (Hoyle & Smith, 1994), we included all subscales in the analyses of the data.

### Statistical Analyses

The self-reported change in behavior was tested against zero using a two-sided z-test to examine whether participants of the study on average perceived a significant change in their behavior in response to the COVID-19 pandemic, and include Cohen’s *d* as measure for effect size.

The relations between the behavioral changes and the potential predictors were tested using a structural equation model. We modelled the CBC subscales, the perceived vulnerability to disease and the general distress (PHQ-4) as latent factors, and included the percentage of correct knowledge about COVID-19 as a manifest indicator. Standardized *β* coefficients are reported for the structural part of the model and serve as effect size measure.

In addition, we compared three groups with a low, medium and high PVD value, based on the low and upper quartiles, with the second and third quartiles forming the moderate PVD group. The groups were compared with regard to knowledge about COVID-19, PHQ-4 and CBC subscales. A MANOVA was conducted to test group differences, with post-hoc ANOVAs and t-tests where necessary. We calculated *η*^2^ as effect size measure for MANOVA and ANOVAs, and Cohen’s *d* for post-hoc t-tests. We applied the Holm correction for multiple tests for the post-hoc t-tests. All calculations were performed with the statistics software R (Core Team, 2020), using the packages lavaan (Rosseel, 2012), BSDA (Arnholt & Evans, 2017) and userfriendlyscience (Peters, 2018).

## Results

### Behavioral Changes

The average self-reported behavioral changes were significantly different from zero for adaptive (*M* = 0.58, *z* = 25.12, *p* < .001, Cohen’s *d* = 0.68), preventive (*M* = 1.53, *z* = 70.48, *p* <. 001, Cohens’s *d* = 1.92) and risk behavior (*M* = −1.66, *z* = −43.92, *p* < .001, Cohen’s *d* = 1.19), which constitute medium to large effects. Means and standard deviations for all scales can be found in Tables 1 and 2. Rather low correlations were found between most of the measured variables.

**Table 1:** Means and standard deviations of the Perceived vulnerability to disease scale (PVD) total, and subscales Perceived Infectability and Germ Aversion, COVID-19 Behavior Checklist (CBC), Knowledge on COVID-19 Questionnaire (CKQ), Patient Health Questionnaire (PHQ-4), and their intercorrelations

	M	SD	2.	3.	4.	5.	6.	7.	8.
1. PVD	3.98	0.97	.888**	.757**	.225**	.099*	- .105*	.253**	−.047
2. Perceived Infectability	3.27	1.46		.303**	.282**	.052	−.041	.170**	−.116*
3. Germ Aversion	4.89	1.11			.136*	.088	.104*	.245**	- .023
4. PHQ-4	1.74	0.79				- .012	- .029	.107*	- .140**
5. CKQ	76.14	11.76					- .041	.086*	.049
6. CBC Risk Behavior	- 1.66	1.39						- .110*	- .030
7. CBC Preventive Behavior	1.53	0.80							0.072
8. CBC Adaptive Behavior	0.58	0.85							

*Note.* **: p < .001, *: *p* < .05. Pearson correlation coefficients used. PVD: Perceived vulnerability to disease Scale, CBC: COVID-19 Behavior Checklist, CKQ: Knowledge on COVID-19, PHQ-4: Patient Health Questionnaire

**Table 2:** Means and standard deviations of COVID-19 Behavior Checklist (CBC), Knowledge on COVID-19 (CKQ), and Patient Health Questionnaire (PHQ-4) for the low-, moderate- and high-PVD group and overall group differences

		Low PVD *N* = 338	Moderate PVD *N* = 673	High PVD *N* = 347	ANOVA
CBC Risk Behavior	M SD	- 1.48 1.32	- 1.66 1.37	- 1.82 1.47	F = 10.48, p = .001, *η*^2^ = 0.01
CBC Preventive Behavior	M SD	1.23 0.81	1.57 0.75	1.73 0.79	F = 69.49, *p* < .001, *η*^2^ = 0.05
CBC Adaptive Behavior	M SD	0.60 0.81	0.60 0.82	0.52 0.95	F = 1.36, *p* = .244
CKQ	M SD	- 0.12 0.99	- 0.02 1.02	0.15 0.96	F = 58.42, p < .001, *η*^2^ = 0.04
PHQ-4	M SD	- 0.19 0.96	- 0.10 0.91	0.15 1.11	F = 11.92, p < .001, *η*^2^ = 0.01

*Note.* PVD: Perceived vulnerability to disease Scale, CBC: COVID-19 Behavior Checklist, CKQ: Knowledge on COVID-19, PHQ-4: Patient Health Questionnaire

### Predictors of Self-Rated Behavioral Change in Response to the Pandemic

We conducted a structural equation model to test the hypotheses specified above about the influencing factors on the behavioral scales.

As hypothesized, knowledge about COVID-19 predicted stronger preventive behavior (*β* = 0.156, *p* = .017) and adaptive behavior (*β* = 0.133, *p* = .032), while no relation was found to risk behavior. General distress (PHQ-4) was found to significantly predict the perceived infectability (*β* = 0.352, *p* < .001) as well as germ aversion (*β* = 0.251, *p* < .001). Higher general distress, additionally, was related to a reported reduction in adaptive behavior (*β* = − 0.162, *p* = .032). Germ aversion predicted a reported increase in preventive (*β* = 0.448, *p* = < .001) and a decrease in risk behavior (*β* = − 0.172, *p* = .013), while the perceived infectability subscale was related to a reported increase in preventive behavior (*β* = 0.158, *p* = .032). Effects are in the small to medium range.

**Fig. 1:**
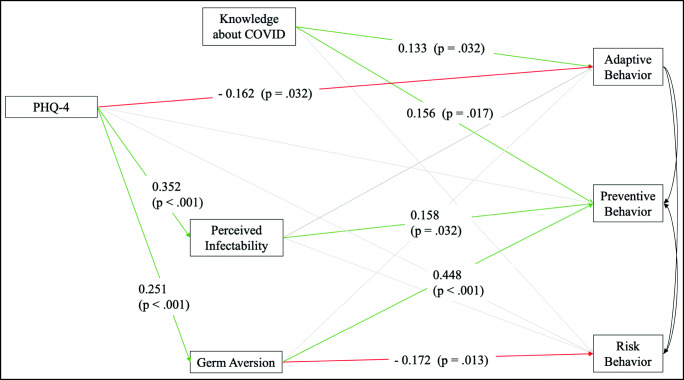
Regressions from the structural equation model on the relationship between Knowledge about COVID-19, Perceived vulnerability to disease, and behavioral changes related to COVID-19. *Note.* Standardized coefficients are presented

**Fig. 2:**
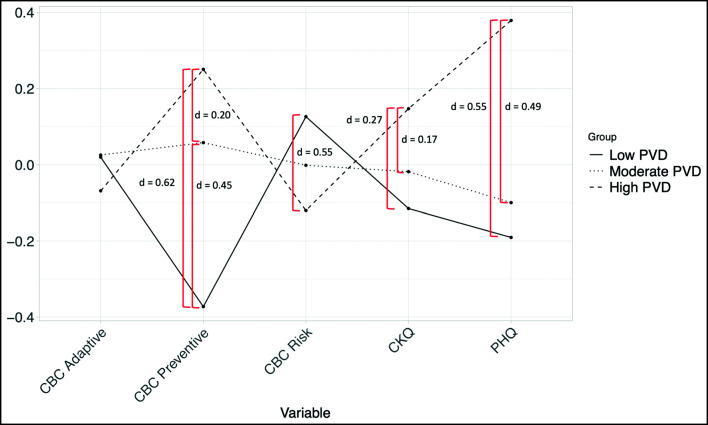
COVID-19 related behaviors, Knowledge about COVID-19 (CKQ), and emotional distress (PHQ-4) in participants with low, medium and high Perceived Vulnerability to Disease. *Note.* z-Standardized values are used for this figure

The relation of general distress to preventive behavior was mediated by the perceived infectability (*p* = .039) and germ aversion (*p* = .004), which is shown by the fact that the significant path coefficient (*model without mediators: β* = .135, *p* < .001) disappeared when the mediators were included (*model with mediators: β* = .044, *p* = .578) (Figs. 1 and 2).

### Comparison of Extreme Groups on Perceived Vulnerability to Disease

The MANOVA indicated a significant group difference over all variables (*F* = 27.26, *p* < .001, *η*^2^ = 0.09). Univariate comparisons between pairs of PVD-related groups were used to allocate group differences more specifically. We found a small, but significant group difference in the change in risk behavior (*F* = 10.48, *p* = .001, *η*^2^ = 0.01), that could be traced back to a difference between the low- and high-PVD group (*t* = 3.21, *p* = .001, Cohen’s *d* = 0.25). A significant, small group difference (*F* = 69.49, *p* < .001, *η*^2^ = 0.05) was found for preventive behavior between all three PVD groups, that ranged from small to large effect size (Low vs. Medium: *t* = −6.50, *p* < .001, Cohen’s *d* = 0.45; Low vs. High: *t* = −8.09, *p* < .001, Cohen’s *d* = 0.62; Medium vs. High: *t* = −2.98, *p* = .002, Cohen’s *d* = 0.20). However, the PVD groups did not differ significantly in their change in adaptive behavior. The comparison of PHQ values showed a small, but significant overall group difference (*F* = 58.42, *p* < .001, *η*^2^ = 0.04), that was traced back to medium large and large effects between the low and the high (*t* = −7.19, *p* < .001, Cohen’s *d* = 0.55) and the medium and high (*t* = −6.94, *p* < .001, Cohen’s *d* = .49) PVD group. CKQ values also differed in the three PVD groups (*F* = 11.92, *p* < .001, *η*^2^ = 0.01), with higher CKQ values in the high PVD group compared to the low PVD (*t* = −3.51, *p* < .001, Cohen’s *d* = 0.27) and medium PVD (*t* = − 2.56, *p* = .011, Cohen’s *d* = 0.17) group, constituting small effects.

## Discussion

The main goal of our study was to explore the relationship of perceived vulnerability to disease and knowledge about COVID-19 to behavioral responses to the current COVID-19 pandemic. To assess behavioral changes during lockdown, we constructed a specific measure comprising three aspects: prevention of contagion, adaptation to social restrictions, and continued social activities associated with risk of infection. A confirmatory factor supported the a priori supposed dimensions of preventive, adaptive and risk behavior.

The results of a structural equation model revealed that PVD subscales and knowledge about COVID-19 were associated with different patterns of self-reported behavioral changes. Whereas higher levels of PVD-subscales perceived infectability and germ aversion were associated with a reported increase in preventive behavior, knowledge about COVID-19 was associated with increased preventive, but also increased adaptive behavior. Only the PVD-subscale germ aversion predicted a reported reduction in risk behavior. Although the coefficients are moderate to small, our results may give some support to the findings of a positive correlation between anxiety, knowledge and preventive behavior (Liu et al., 2020; Lei et al., 2020). It should be noted that PVD is suggested to represent a dispositional trait strongly related to infections and may be highly correlated with fear of COVID-19 infection, but we did not use a specific measure to prove this. Nevertheless, Taylor et al. (2020a) found moderate to high correlations of the two PVD subscales with the total score of the COVID Stress Scales which measures the COVID stress syndrome.

We also found significant, though low correlations of PVD with symptoms of clinically relevant emotional distress, as assessed by the PHQ-4. In our sample, more than half of the participants (57%) reported scores exceeding the cut-off value for clinically relevant symptoms of depression and anxiety (Löwe et al., 2010). This proportion is much higher than in a larger convenience sample in Germany (25%), recruited under similar conditions (Petzold et al., 2020). In contrast to this high number of participants with symptoms of emotional distress, only a small proportion (12.7%) reported being currently treated in psychotherapy. This proportion is somewhat lower (12.7 vs. 17.9%) than the rate obtained in the study by Asmundson, Paluszek, Landry, McKay, and Taylor (2020) though the latter included also pre-existing disorders (i.e. past years). The discrepancy between high PHQ-8 values and self-reported mental disorders in our study may indicate that many participants experienced an increase of symptoms of depression and anxiety due to the lockdown not related to a current mental disorder. Unfortunately, we did not assess mental disorders during the past year, which might also be worse during lockdown (Asmundson et al., 2020).

Actually, symptoms of depression or anxiety moderated the impact of PVD on adaptive behavioral reactions to COVID-19 pandemic, e.g. calling friends, walking or doing sports. Regression analyses showed that knowledge is associated with increased adaptive behavior in participants with higher PHQ-4 values, which suggests that participants attempt to regulate their high level of emotional distress. However, this interaction effect was very low, and may lack practical significance. More consistent with our expectations, the association between PVD and adaptive behavior was stronger when PHQ-4 scores are low than when they are high. Although there was a significant correlation between PVD and emotional distress, adaptive coping with the psychological and social threats of the pandemic may be enhanced if individuals can regulate anxiety and depression, despite high levels of fear of infection (Jungmann & Witthöft, 2020). However, as with any cross-sectional association, it is not possible to determine the direction of causality of the reported relationship.

The importance of perceived vulnerability of distress is illustrated by the comparison of extreme groups (low vs. moderate vs. high) on the basis of lower and upper quartiles. The high-PVD group showed not only significantly more preventive behavior, but showed also significantly more knowledge and more emotional distress than the moderate- and low-PVD groups. In addition, the high and the low PVD group differed in their ratings of change in risk behavior. The low-PVD group differed from the medium-PVD group also in terms of less increase in preventive behavior. Thus, the low-PVD group showed unfavorable reactions to the pandemic, despite the strong recommendation by health authorities. It should be noted, however, that health anxiety represents a continuum ranging from an absence of health concerns to pathological health anxiety, and that dimensional approaches have clear methodological advantages over categorial taxonomies (Ferguson, 2009). Nevertheless, the results illustrate a pattern of cognitive, affective and behavioral response that may be helpful to interpret in relationship to the concept of the behavioral immune system (Schaller & Park, 2011; Sawada, Auger, & Lydon, 2018; Taylor, 2019).

Within this theoretical context, preventive behaviors may represent a psychological “first line of defense” system that prompts avoidance behavior to promote physical health, but at the expense of mental health. In the trade-off between social gains and potential risk of infection, low-PVD individuals may prefer to maintain their wellbeing even if this is at the expense of their physical health. In contrast, high-PVD individuals may exhibit a stronger avoidance motivation in order to protect the self from disease (Sawada et al., 2018; Shakar, 2019). Since the knowledge about the modes of transmission of COVID-19 was small during the beginning of the pandemic, the lack of controllability and predictability has been associated with much uncertainty. Under these circumstances, competing basic motivational systems may be triggered, resulting in a preference either for the motivation to minimize risk of disease, i.e. activation of the behavioral immune system, or for the motivation to maximize/maintain social gains, e.g. satisfying the need for belonging/attachment (Sawada et al., 2018).

Interestingly, the mean PVD-score of our sample is nearly identical to the PVDQ score of a Japanese online-survey with comparable sociodemographic characteristics which was conducted before the COVID-19 pandemic in 2019 (Yamada, Xu, & Sasaki, 2020). This unexpected result allows for several interpretations. First, the pandemic may lead to an increase of PVD, but the samples may differ due to different mechanisms of self-selection, e.g. with more PVD-sensitive participants being selected in the Japanese sample. Second, PVD may be a stable trait or disposition which is not influenced by current pandemics. Third, cultural differences such as East Asian vs. European coping patterns facing pandemics, or cultural values such as individualism vs. collectivism, may have compensated for a possible impact of the pandemic in Germany, as compared to the pre-COVID-19 situation in Japan. For instance, Skolnick and Dzokoto (2013) found that the PVD scores were significantly higher in Ghana, a country with a historically high prevalence of infectious diseases, than in the United States with lower levels of disease threat. Future studies should be conducted longitudinally in different countries and stages of the pandemic to clarify these questions.

There are several limitations to this study. First, our sample was not representative of the German general population in terms of gender (76% female) and education (82,8% Bachelor’s or Master’s degree). Furthermore, despite using different sources of recruitment without participant compensation, the self-selection of our convenience sample probably favors individuals interested in psychological issues. It is possible that the online survey might have been completed by individuals with higher average PVD scores than the general population. This assumption is supported by the high rate of participants with elevated PHQ-4 scores, which was significantly increased in comparison to the rate in the general population (Löwe et al., 2010). However, it should be noted that this measure is based on a ultra-brief four-item measure of depression and anxiety, with acceptable reliability and construct validity, but restricted validity with respect to mental disorders.

A second limitation is given by the use of non-standardized measures for knowledge and COVID-19 related behavioral changes. Although we used a multiple-choice test format that allowed for an objective evaluation of knowledge, selected rather specific behaviors for the COVID-19 behavior checklist and checked factorial validity, more information is required on the external validity of both measures. Third, the behavioral scales as well as the PVD subscales show low internal consistencies. Therefore, we decided to include these scales at latent factors in a SEM framework to compensate for the low internal consistency. In latent modeling, true variance is separated from error variance and only true variance portions are used to calculate the relationships, which increases the reliability of the regressions. However, more data are needed to support the validity of the risk and adaptive behavior subscales. For instance, ecological momentary assessment of relevant behaviors may compensate for the methodological weaknesses of the retrospective assessment in an online survey.

Forth, we did not include Health Locus of Control because this construct has been found relevant for the prediction of general health behavior (e.g. Helmer, Krämer, & Mikolajczyk, 2012), but not of preventive behaviors relevant for COVID-19 pandemic (Pagnini et al., 2020). A probable reason may be that internal locus of control is relevant for general indicators of health maintenance behaviour (e.g. nutrition), but may be less predictive for behaviors when facing specific health threats (Steptoe & Wardle, 2001).

Fifth, we did not use a specific measure for fear of COVID-19, which was not available at the beginning of the study. The inclusion of standardized measures, such as the “COVID Stress Scales” (Taylor et al., 2020b) or the “Fear of COVID Scale” (Ahorsu et al. 2020) would offer the possibility to discriminate more accurately between dispositional aspects of fear of infection, and specific fears of COVID-19 infection. Finally, our cross-sectional data do not allow for reliable conclusions concerning the causal relationship between cognitive, emotional and behavioral reactions to a rapidly changing pandemic situation such as during the pandemic lockdown. Another limitation may be the assessment of behavioral change at an early phase of the pandemic. In response to COVID-19, risk, adaptive, and preventive behaviors may vary throughout the phases of behavior change (Frissen et al., 2020). Ongoing studies should evaluate behavioral change depending on different stages of the COVID-19 pandemic and other factors than motivation, such as perceived benefits and barriers of health behavior as described in the Health Belief Model (Rosenstock et al., 1988; Jones et al., 2014).

Nonetheless, the study provides important preliminary findings on the relationship between the perception of disease as a dispositional measure, knowledge and emotional distress on important indicators of the behavioral immune system. In particular, perceived vulnerability to disease and emotional distress contributed to the prediction of preventive behavior. However, future studies should compensate for the methodological weaknesses by developing reliable instruments, using representative samples and including further dimensions from research on health behavior which may be relevant to cope with the COVID-19 pandemic.
